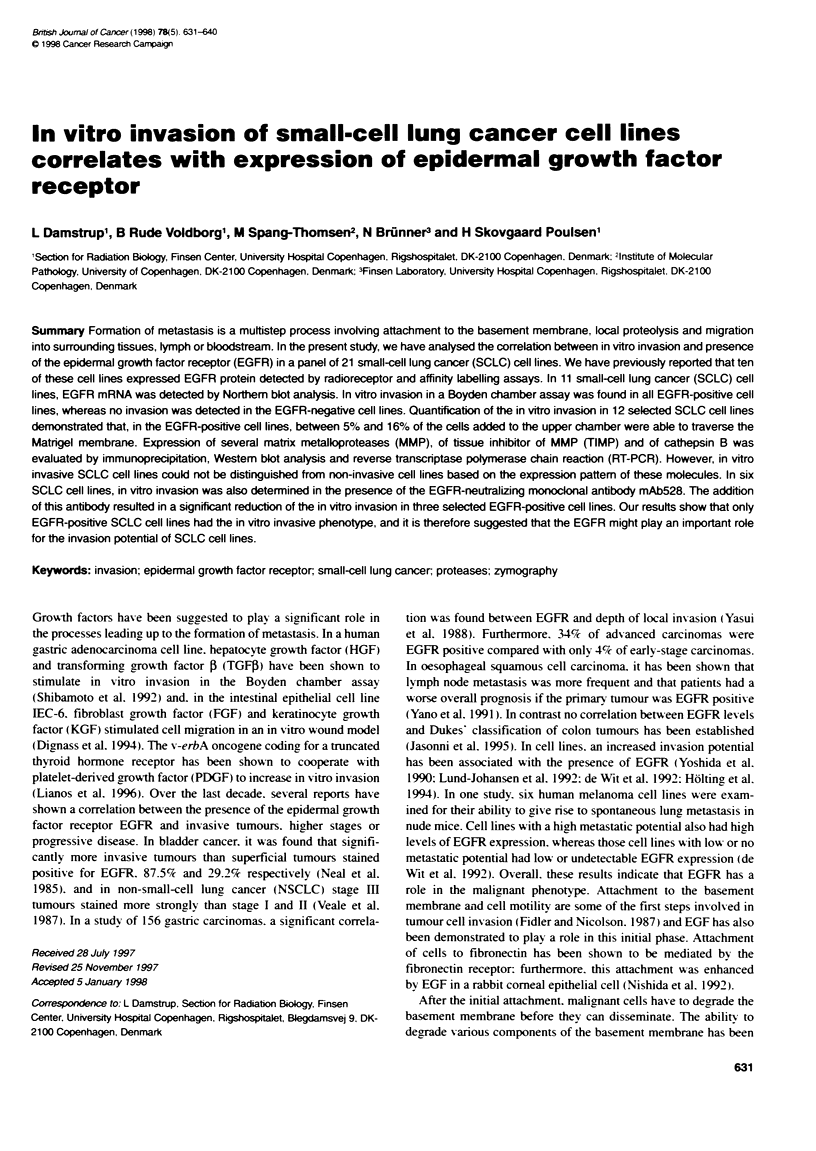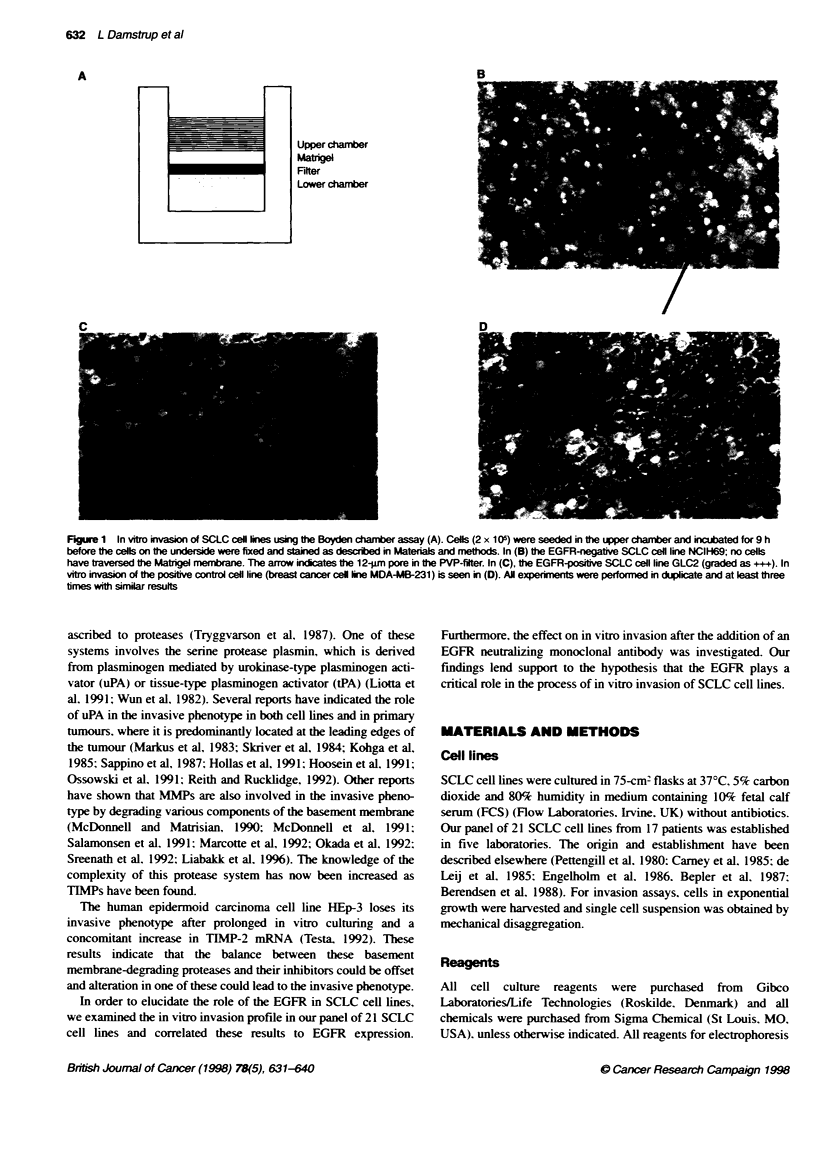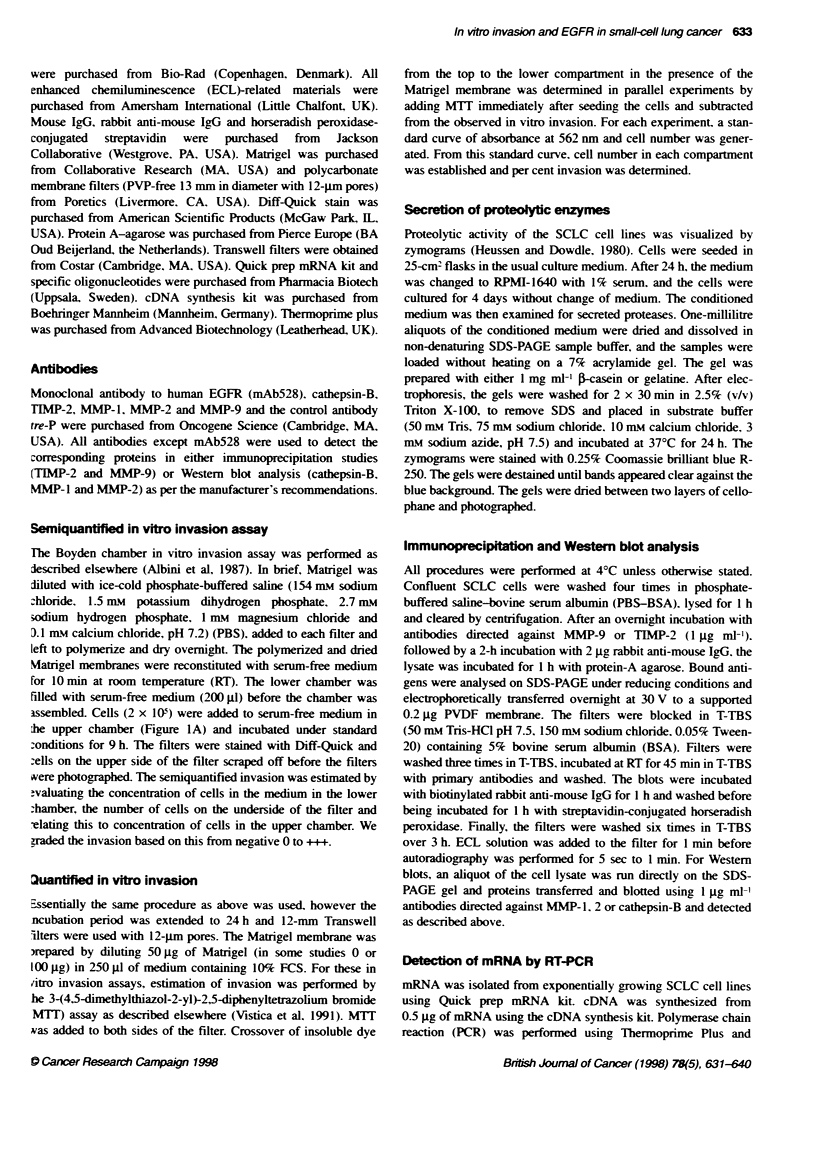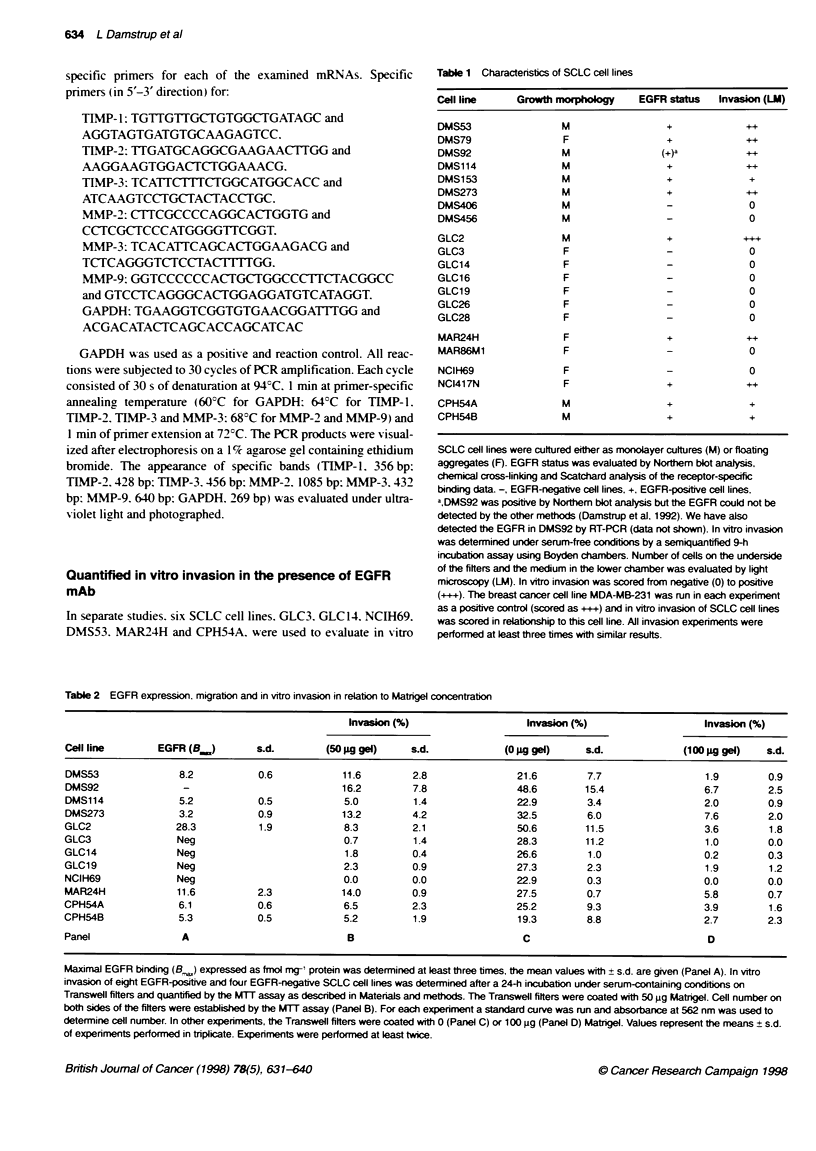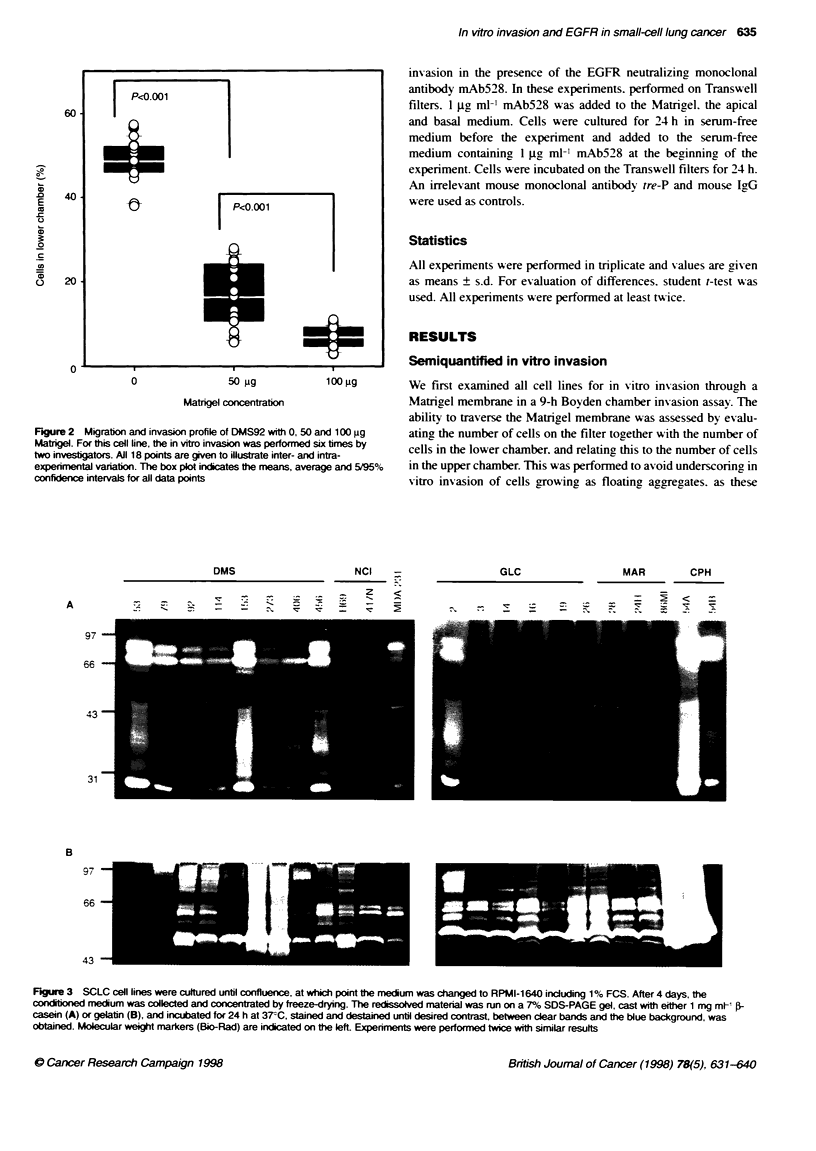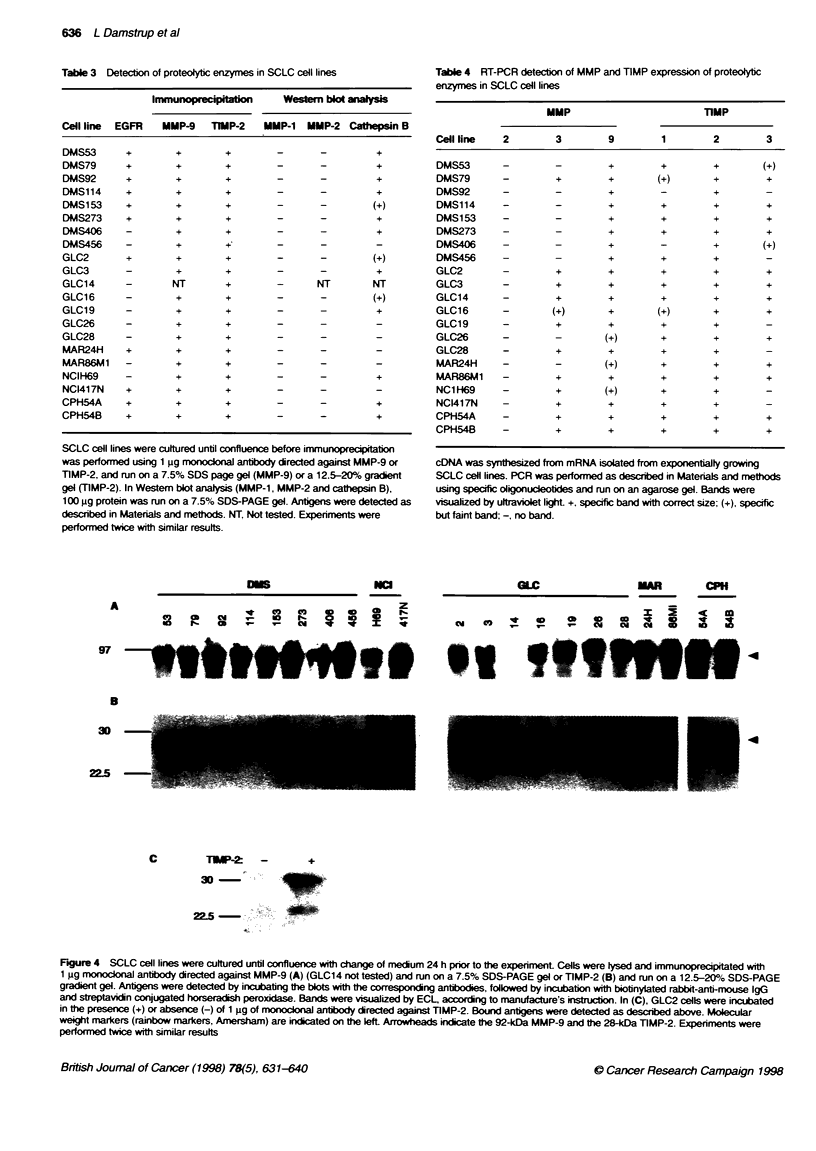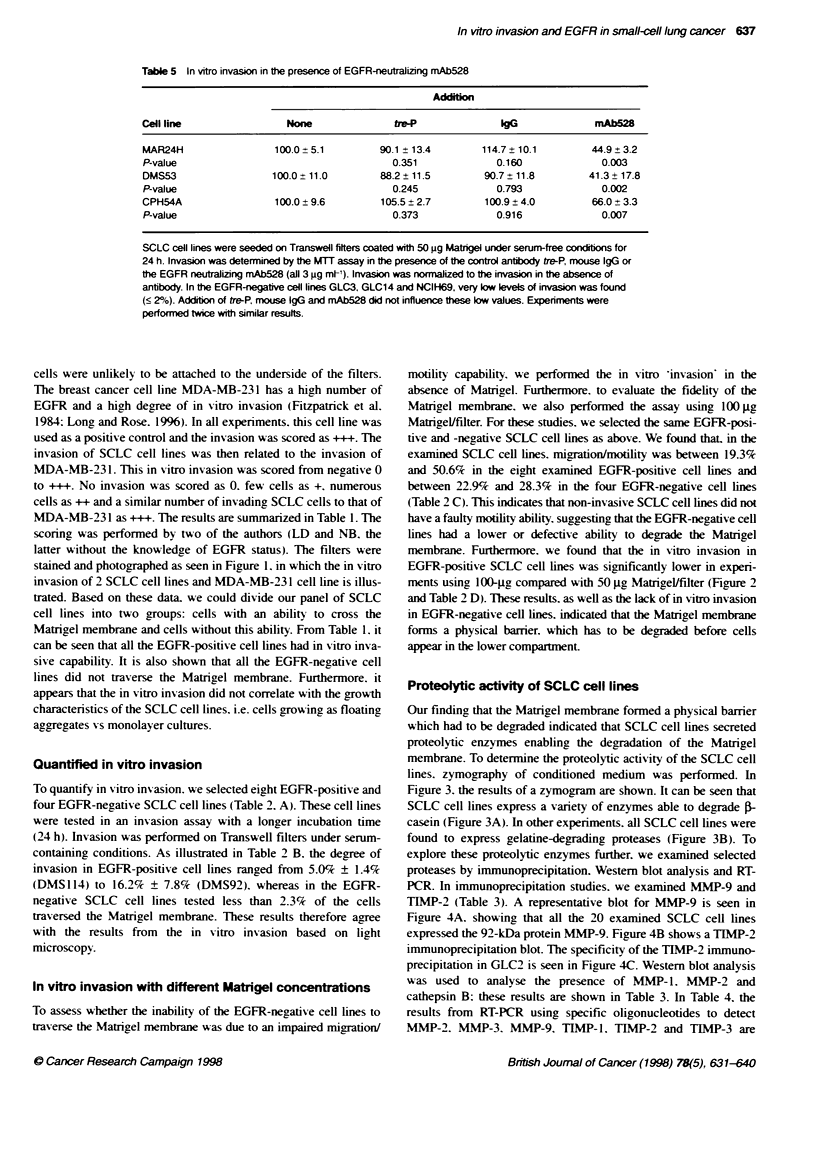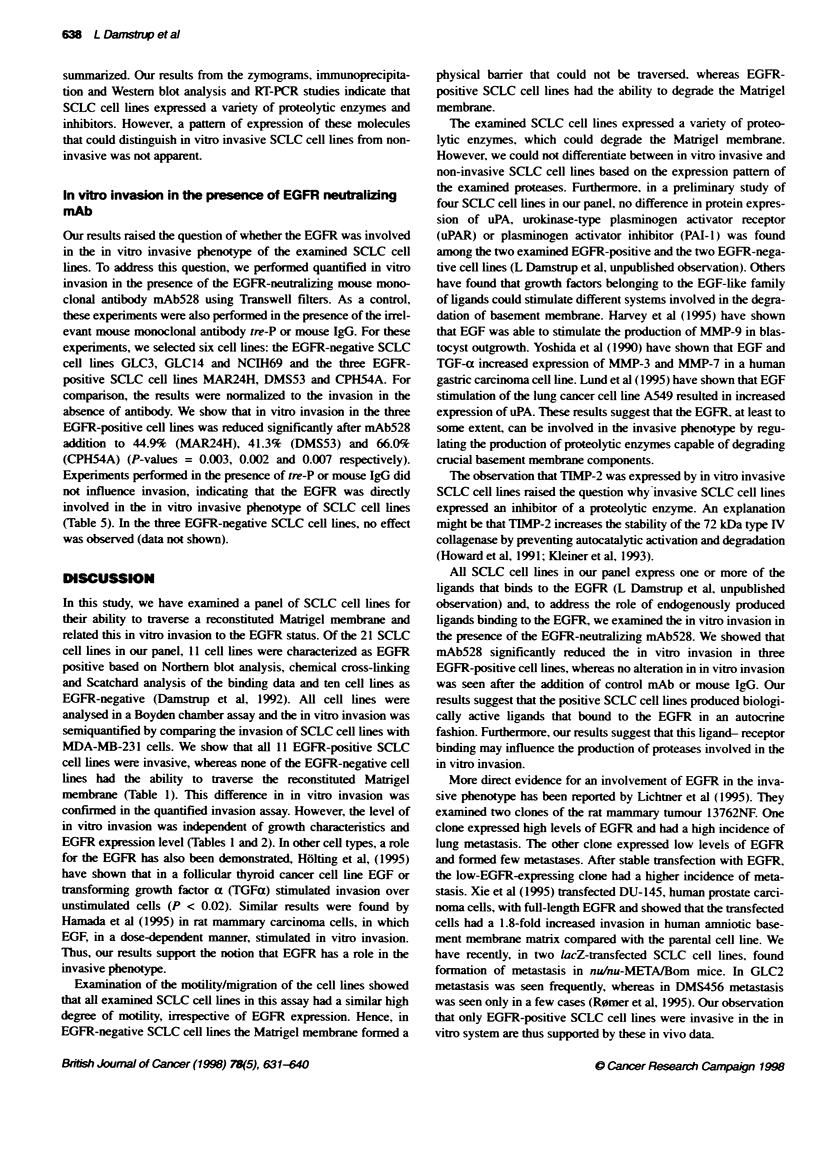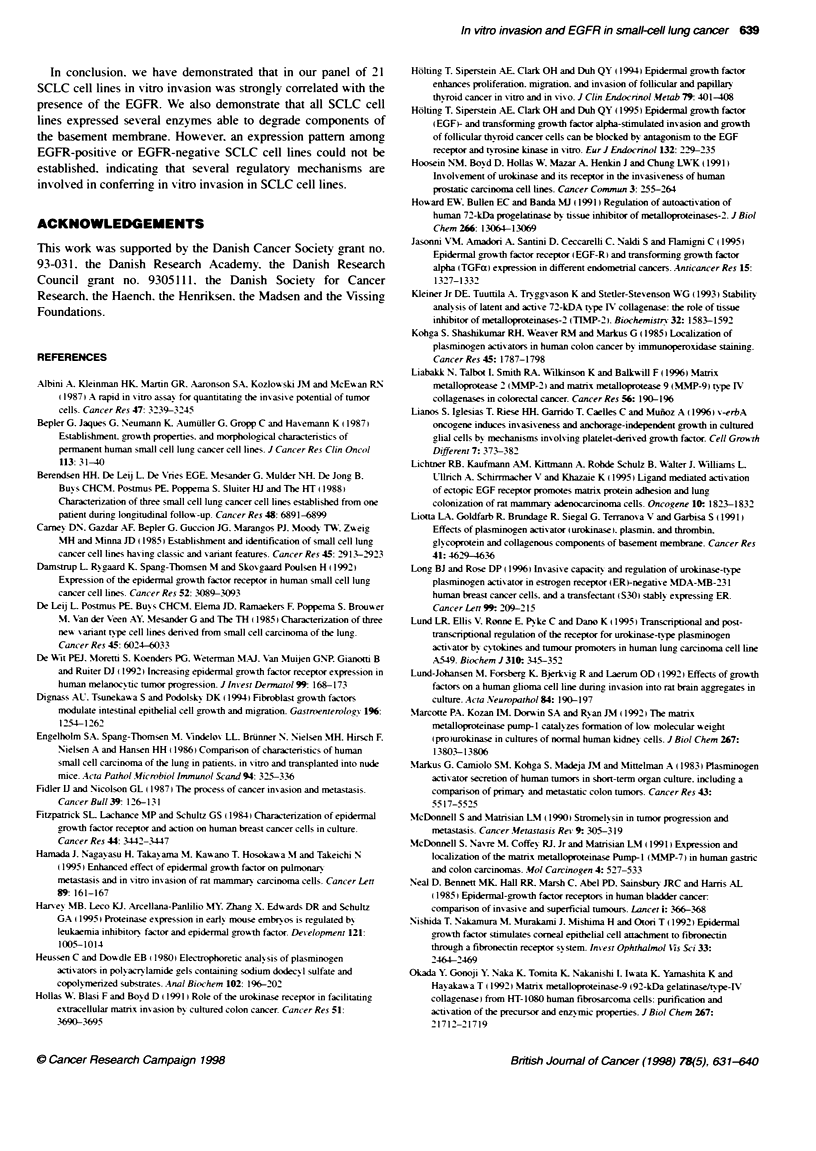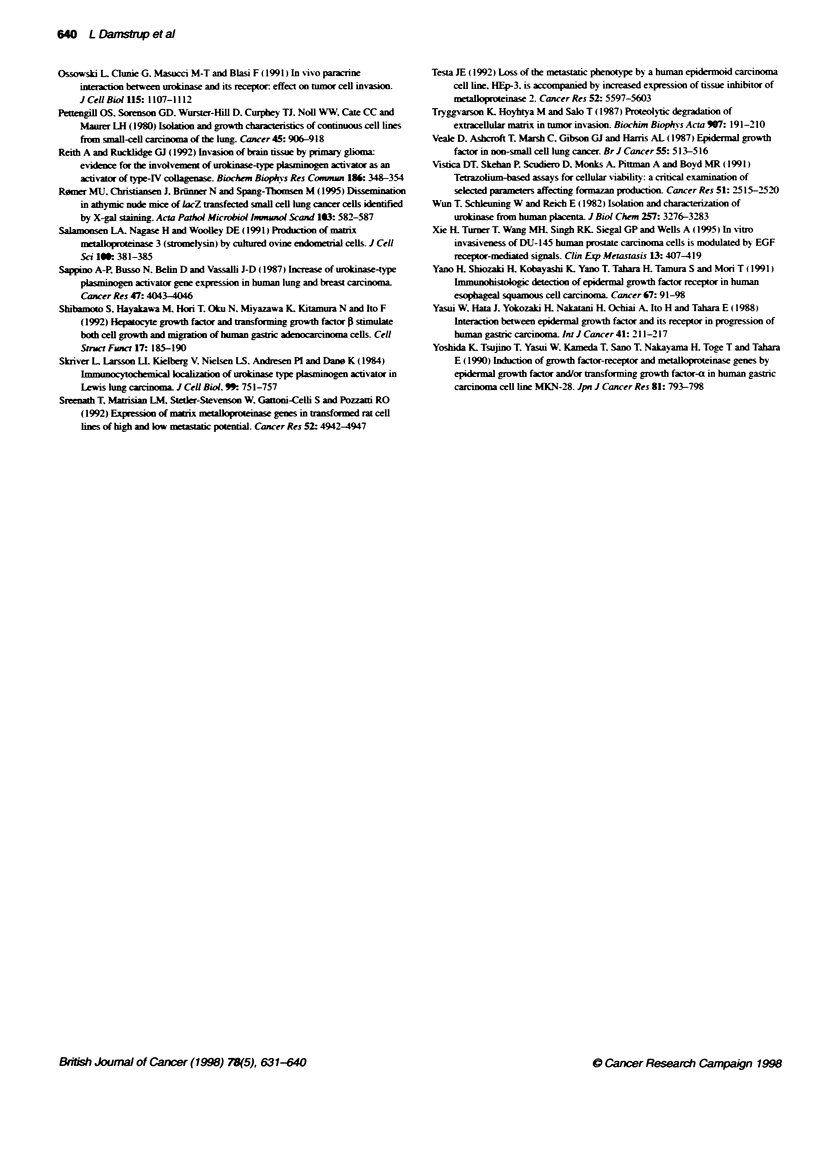# In vitro invasion of small-cell lung cancer cell lines correlates with expression of epidermal growth factor receptor.

**DOI:** 10.1038/bjc.1998.553

**Published:** 1998-09

**Authors:** L. Damstrup, B. Rude Voldborg, M. Spang-Thomsen, N. Brünner, H. Skovgaard Poulsen

**Affiliations:** Section for Radiation Biology, Finsen Center, University Hospital Copenhagen, Rigshospitalet, Denmark.

## Abstract

**Images:**


British Journal of Cancer (1998) 78(5). 631–640
© 1998 Cancer Research Campaign

### In vitro invasion of small-cell lung cancer cell lines correlates with expression of epidermal growth factor receptor

**L Damstrup[1], B Rude Voldborg[1], M Spang-Thomsen[2], N Brünner[3] and H Skovgaard Poulsen[1]**

[1]Section for Radiation Biology, Finsen Center, University Hospital Copenhagen, Rigshospitalet, DK-2100 Copenhagen, Denmark; [2]Institute of Molecular Pathology, University of Copenhagen, DK-2100 Copenhagen, Denmark; [3]Finsen Laboratory, University Hospital Copenhagen, Rigshospitalet, DK-2100 Copenhagen, Denmark

**Summary** Formation of metastasis is a multistep process involving attachment to the basement membrane, local proteolysis and migration into surrounding tissues, lymph or bloodstream. In the present study, we have analysed the correlation between in vitro invasion and presence of the epidermal growth factor receptor (EGFR) in a panel of 21 small-cell lung cancer (SCLC) cell lines. We have previously reported that ten of these cell lines expressed EGFR protein detected by radioreceptor and affinity labelling assays. In 11 small-cell lung cancer (SCLC) cell lines, EGFR mRNA was detected by Northern blot analysis. In vitro invasion in a Boyden chamber assay was found in all EGFR-positive cell lines, whereas no invasion was detected in the EGFR-negative cell lines. Quantification of the in vitro invasion in 12 selected SCLC cell lines demonstrated that, in the EGFR-positive cell lines, between 5% and 16% of the cells added to the upper chamber were able to traverse the Matrigel membrane. Expression of several matrix metalloproteases (MMP), of tissue inhibitor of MMP (TIMP) and of cathepsin B was evaluated by immunoprecipitation, Western blot analysis and reverse transcriptase polymerase chain reaction (RT-PCR). However, in vitro invasive SCLC cell lines could not be distinguished from non-invasive cell lines based on the expression pattern of these molecules. In six SCLC cell lines, in vitro invasion was also determined in the presence of the EGFR-neutralizing monoclonal antibody mAb528. The addition of this antibody resulted in a significant reduction of the in vitro invasion in three selected EGFR-positive cell lines. Our results show that only EGFR-positive SCLC cell lines had the in vitro invasive phenotype, and it is therefore suggested that the EGFR might play an important role for the invasion potential of SCLC cell lines.

**Keywords:** invasion; epidermal growth factor receptor; small-cell lung cancer; proteases; zymography

Growth factors have been suggested to play a significant role in the processes leading up to the formation of metastasis. In a human gastric adenocarcinoma cell line, hepatocyte growth factor (HGF) and transforming growth factor β (TGFβ) have been shown to stimulate in vitro invasion in the Boyden chamber assay (Shibamoto et al, 1992) and, in the intestinal epithelial cell line IEC-6, fibroblast growth factor (FGF) and keratinocyte growth factor (KGF) stimulated cell migration in an in vitro wound model (Dignass et al, 1994). The v-*erb*A oncogene coding for a truncated thyroid hormone receptor has been shown to cooperate with platelet-derived growth factor (PDGF) to increase in vitro invasion (Lianos et al, 1996). Over the last decade, several reports have shown a correlation between the presence of the epidermal growth factor receptor EGFR and invasive tumours, higher stages or progressive disease. In bladder cancer, it was found that significantly more invasive tumours than superficial tumours stained positive for EGFR, 87.5% and 29.2% respectively (Neal et al, 1985), and in non-small-cell lung cancer (NSCLC) stage III tumours stained more strongly than stage I and II (Veale et al, 1987). In a study of 156 gastric carcinomas, a significant correlation was found between EGFR and depth of local invasion (Yasui et al, 1988). Furthermore, 34% of advanced carcinomas were EGFR positive compared with only 4% of early-stage carcinomas. In oesophageal squamous cell carcinoma, it has been shown that lymph node metastasis was more frequent and that patients had a worse overall prognosis if the primary tumour was EGFR positive (Yano et al, 1991). In contrast no correlation between EGFR levels and Dukes' classification of colon tumours has been established (Jasonni et al, 1995). In cell lines, an increased invasion potential has been associated with the presence of EGFR (Yoshida et al, 1990; Lund-Johansen et al, 1992; de Wit et al, 1992; Hölting et al, 1994). In one study, six human melanoma cell lines were examined for their ability to give rise to spontaneous lung metastasis in nude mice. Cell lines with a high metastatic potential also had high levels of EGFR expression, whereas those cell lines with low or no metastatic potential had low or undetectable EGFR expression (de Wit et al, 1992). Overall, these results indicate that EGFR has a role in the malignant phenotype. Attachment to the basement membrane and cell motility are some of the first steps involved in tumour cell invasion (Fidler and Nicolson, 1987) and EGF has also been demonstrated to play a role in this initial phase. Attachment of cells to fibronectin has been shown to be mediated by the fibronectin receptor; furthermore, this attachment was enhanced by EGF in a rabbit corneal epithelial cell (Nishida et al, 1992).

After the initial attachment, malignant cells have to degrade the basement membrane before they can disseminate. The ability to degrade various components of the basement membrane has been

*Received 28 July 1997*
*Revised 25 November 1997*
*Accepted 5 January 1998*

*Correspondence to:* L Damstrup, Section for Radiation Biology, Finsen Center, University Hospital Copenhagen, Rigshospitalet, Blegdamsvej 9, DK-2100 Copenhagen, Denmark

**Figure 1** In vitro invasion of SCLC cell lines using the Boyden chamber assay (A). Cells (2 × $10^5$) were seeded in the upper chamber and incubated for 9 h before the cells on the underside were fixed and stained as described in Materials and methods. In (B) the EGFR-negative SCLC cell line NCIH69; no cells have traversed the Matrigel membrane. The arrow indicates the 12-μm pore in the PVP-filter. In (C), the EGFR-positive SCLC cell line GLC2 (graded as +++). In vitro invasion of the positive control cell line (breast cancer cell line MDA-MB-231) is seen in (D). All experiments were performed in duplicate and at least three times with similar results ascribed to proteases (Tryggvarson et al, 1987). One of these systems involves the serine protease plasmin, which is derived from plasminogen mediated by urokinase-type plasminogen activator (uPA) or tissue-type plasminogen activator (tPA) (Liotta et al, 1991; Wun et al, 1982). Several reports have indicated the role of uPA in the invasive phenotype in both cell lines and in primary tumours, where it is predominantly located at the leading edges of the tumour (Markus et al, 1983; Skriver et al, 1984; Kohga et al, 1985; Sappino et al, 1987; Hollas et al, 1991; Hoosein et al, 1991; Ossowski et al, 1991; Reith and Rucklidge, 1992). Other reports have shown that MMPs are also involved in the invasive phenotype by degrading various components of the basement membrane (McDonnell and Matrisian, 1990; McDonnell et al, 1991; Salamonsen et al, 1991; Marcotte et al, 1992; Okada et al, 1992; Sreenath et al, 1992; Liabakk et al, 1996). The knowledge of the complexity of this protease system has now been increased as TIMPs have been found.

The human epidermoid carcinoma cell line HEp-3 loses its invasive phenotype after prolonged in vitro culturing and a concomitant increase in TIMP-2 mRNA (Testa, 1992). These results indicate that the balance between these basement membrane-degrading proteases and their inhibitors could be offset and alteration in one of these could lead to the invasive phenotype.

In order to elucidate the role of the EGFR in SCLC cell lines, we examined the in vitro invasion profile in our panel of 21 SCLC cell lines and correlated these results to EGFR expression. Furthermore, the effect on in vitro invasion after the addition of an EGFR neutralizing monoclonal antibody was investigated. Our findings lend support to the hypothesis that the EGFR plays a critical role in the process of in vitro invasion of SCLC cell lines.

### MATERIALS AND METHODS

### Cell lines

SCLC cell lines were cultured in 75-$cm^2$ flasks at 37°C, 5% carbon dioxide and 80% humidity in medium containing 10% fetal calf serum (FCS) (Flow Laboratories, Irvine, UK) without antibiotics. Our panel of 21 SCLC cell lines from 17 patients was established in five laboratories. The origin and establishment have been described elsewhere (Pettengill et al, 1980; Carney et al, 1985; de Leij et al, 1985; Engelholm et al, 1986, Bepler et al, 1987; Berendsen et al, 1988). For invasion assays, cells in exponential growth were harvested and single cell suspension was obtained by mechanical disaggregation.

### Reagents

All cell culture reagents were purchased from Gibco Laboratories/Life Technologies (Roskilde, Denmark) and all chemicals were purchased from Sigma Chemical (St Louis, MO, USA), unless otherwise indicated. All reagents for electrophoresis

 © Cancer Research Campaign 1998

were purchased from Bio-Rad (Copenhagen, Denmark). All enhanced chemiluminescence (ECL)-related materials were purchased from Amersham International (Little Chalfont, UK). Mouse IgG, rabbit anti-mouse IgG and horseradish peroxidase-conjugated streptavidin were purchased from Jackson Collaborative (Westgrove, PA, USA). Matrigel was purchased from Collaborative Research (MA, USA) and polycarbonate membrane filters (PVP-free 13 mm in diameter with 12-μm pores) from Poretics (Livermore, CA, USA). Diff-Quick stain was purchased from American Scientific Products (McGaw Park, IL, USA). Protein A–agarose was purchased from Pierce Europe (BA Oud Beijerland, the Netherlands). Transwell filters were obtained from Costar (Cambridge, MA, USA). Quick prep mRNA kit and specific oligonucleotides were purchased from Pharmacia Biotech (Uppsala, Sweden). cDNA synthesis kit was purchased from Boehringer Mannheim (Mannheim, Germany). Thermoprime plus was purchased from Advanced Biotechnology (Leatherhead, UK).

### Antibodies

Monoclonal antibody to human EGFR (mAb528), cathepsin-B, TIMP-2, MMP-1, MMP-2 and MMP-9 and the control antibody *tre*-P were purchased from Oncogene Science (Cambridge, MA, USA). All antibodies except mAb528 were used to detect the corresponding proteins in either immunoprecipitation studies (TIMP-2 and MMP-9) or Western blot analysis (cathepsin-B, MMP-1 and MMP-2) as per the manufacturer's recommendations.

### Semiquantified in vitro invasion assay

The Boyden chamber in vitro invasion assay was performed as described elsewhere (Albini et al, 1987). In brief, Matrigel was diluted with ice-cold phosphate-buffered saline (154 mM sodium chloride, 1.5 mM potassium dihydrogen phosphate, 2.7 mM sodium hydrogen phosphate, 1 mM magnesium chloride and 0.1 mM calcium chloride, pH 7.2) (PBS), added to each filter and left to polymerize and dry overnight. The polymerized and dried Matrigel membranes were reconstituted with serum-free medium for 10 min at room temperature (RT). The lower chamber was filled with serum-free medium (200 μl) before the chamber was assembled. Cells ($2 \times 10^5$) were added to serum-free medium in the upper chamber (Figure 1A) and incubated under standard conditions for 9 h. The filters were stained with Diff-Quick and cells on the upper side of the filter scraped off before the filters were photographed. The semiquantified invasion was estimated by evaluating the concentration of cells in the medium in the lower chamber, the number of cells on the underside of the filter and relating this to concentration of cells in the upper chamber. We graded the invasion based on this from negative 0 to +++.

### Quantified in vitro invasion

Essentially the same procedure as above was used, however the incubation period was extended to 24 h and 12-mm Transwell filters were used with 12-μm pores. The Matrigel membrane was prepared by diluting 50 μg of Matrigel (in some studies 0 or 100 μg) in 250 μl of medium containing 10% FCS. For these in vitro invasion assays, estimation of invasion was performed by the 3-(4,5-dimethylthiazol-2-yl)-2,5-diphenyltetrazolium bromide (MTT) assay as described elsewhere (Vistica et al, 1991). MTT was added to both sides of the filter. Crossover of insoluble dye from the top to the lower compartment in the presence of the Matrigel membrane was determined in parallel experiments by adding MTT immediately after seeding the cells and subtracted from the observed in vitro invasion. For each experiment, a standard curve of absorbance at 562 nm and cell number was generated. From this standard curve, cell number in each compartment was established and per cent invasion was determined.

### Secretion of proteolytic enzymes

Proteolytic activity of the SCLC cell lines was visualized by zymograms (Heussen and Dowdle, 1980). Cells were seeded in 25-cm$^2$ flasks in the usual culture medium. After 24 h, the medium was changed to RPMI-1640 with 1% serum, and the cells were cultured for 4 days without change of medium. The conditioned medium was then examined for secreted proteases. One-millilitre aliquots of the conditioned medium were dried and dissolved in non-denaturing SDS-PAGE sample buffer, and the samples were loaded without heating on a 7% acrylamide gel. The gel was prepared with either 1 mg ml$^{-1}$ β-casein or gelatine. After electrophoresis, the gels were washed for $2 \times 30$ min in 2.5% (v/v) Triton X-100, to remove SDS and placed in substrate buffer (50 mM Tris, 75 mM sodium chloride, 10 mM calcium chloride, 3 mM sodium azide, pH 7.5) and incubated at 37°C for 24 h. The zymograms were stained with 0.25% Coomassie brilliant blue R-250. The gels were destained until bands appeared clear against the blue background. The gels were dried between two layers of cellophane and photographed.

### Immunoprecipitation and Western blot analysis

All procedures were performed at 4°C unless otherwise stated. Confluent SCLC cells were washed four times in phosphate-buffered saline–bovine serum albumin (PBS–BSA), lysed for 1 h and cleared by centrifugation. After an overnight incubation with antibodies directed against MMP-9 or TIMP-2 (1 μg ml$^{-1}$), followed by a 2-h incubation with 2 μg rabbit anti-mouse IgG, the lysate was incubated for 1 h with protein-A agarose. Bound antigens were analysed on SDS-PAGE under reducing conditions and electrophoretically transferred overnight at 30 V to a supported 0.2 μg PVDF membrane. The filters were blocked in T-TBS (50 mM Tris-HCl pH 7.5, 150 mM sodium chloride, 0.05% Tween-20) containing 5% bovine serum albumin (BSA). Filters were washed three times in T-TBS, incubated at RT for 45 min in T-TBS with primary antibodies and washed. The blots were incubated with biotinylated rabbit anti-mouse IgG for 1 h and washed before being incubated for 1 h with streptavidin-conjugated horseradish peroxidase. Finally, the filters were washed six times in T-TBS over 3 h. ECL solution was added to the filter for 1 min before autoradiography was performed for 5 sec to 1 min. For Western blots, an aliquot of the cell lysate was run directly on the SDS-PAGE gel and proteins transferred and blotted using 1 μg ml$^{-1}$ antibodies directed against MMP-1, 2 or cathepsin-B and detected as described above.

### Detection of mRNA by RT-PCR

mRNA was isolated from exponentially growing SCLC cell lines using Quick prep mRNA kit. cDNA was synthesized from 0.5 μg of mRNA using the cDNA synthesis kit. Polymerase chain reaction (PCR) was performed using Thermoprime Plus and

© Cancer Research Campaign 1998 

specific primers for each of the examined mRNAs. Specific primers (in 5′–3′ direction) for:

TIMP-1: TGTTGTTGCTGTGGCTGATAGC and AGGTAGTGATGTGCAAGAGTCC.
TIMP-2: TTGATGCAGGCGAAGAACTTGG and AAGGAAGTGGACTCTGGAAACG.
TIMP-3: TCATTCTTTCTGGCATGGCACC and ATCAAGTCCTGCTACTACCTGC.
MMP-2: CTTCGCCCCAGGCACTGGTG and CCTCGCTCCCATGGGGTTCGGT.
MMP-3: TCACATTCAGCACTGGAAGACG and TCTCAGGGTCTCCTACTTTTGG.
MMP-9: GGTCCCCCCACTGCTGGCCCTTCTACGGCC and GTCCTCAGGGCACTGGAGGATGTCATAGGT.
GAPDH: TGAAGGTCGGTGTGAACGGATTTGG and ACGACATACTCAGCACCAGCATCAC

GAPDH was used as a positive and reaction control. All reactions were subjected to 30 cycles of PCR amplification. Each cycle consisted of 30 s of denaturation at 94°C, 1 min at primer-specific annealing temperature (60°C for GAPDH; 64°C for TIMP-1, TIMP-2, TIMP-3 and MMP-3; 68°C for MMP-2 and MMP-9) and 1 min of primer extension at 72°C. The PCR products were visualized after electrophoresis on a 1% agarose gel containing ethidium bromide. The appearance of specific bands (TIMP-1, 356 bp; TIMP-2, 428 bp; TIMP-3, 456 bp; MMP-2, 1085 bp; MMP-3, 432 bp; MMP-9, 640 bp; GAPDH, 269 bp) was evaluated under ultraviolet light and photographed.

### Quantified in vitro invasion in the presence of EGFR mAb

In separate studies, six SCLC cell lines, GLC3, GLC14, NCIH69, DMS53, MAR24H and CPH54A, were used to evaluate in vitro

**Table 1** Characteristics of SCLC cell lines

| Cell line | Growth morphology | EGFR status | Invasion (LM) |
|---|---|---|---|
| DMS53 | M | + | ++ |
| DMS79 | F | + | ++ |
| DMS92 | M | (+)[a] | ++ |
| DMS114 | M | + | ++ |
| DMS153 | M | + | + |
| DMS273 | M | + | ++ |
| DMS406 | M | – | 0 |
| DMS456 | M | – | 0 |
| GLC2 | M | + | +++ |
| GLC3 | F | – | 0 |
| GLC14 | F | – | 0 |
| GLC16 | F | – | 0 |
| GLC19 | F | – | 0 |
| GLC26 | F | – | 0 |
| GLC28 | F | – | 0 |
| MAR24H | F | + | ++ |
| MAR86M1 | F | – | 0 |
| NCIH69 | F | – | 0 |
| NCI417N | F | + | ++ |
| CPH54A | M | + | + |
| CPH54B | M | + | + |

SCLC cell lines were cultured either as monolayer cultures (M) or floating aggregates (F). EGFR status was evaluated by Northern blot analysis, chemical cross-linking and Scatchard analysis of the receptor-specific binding data. –, EGFR-negative cell lines, +, EGFR-positive cell lines. [a],DMS92 was positive by Northern blot analysis but the EGFR could not be detected by the other methods (Damstrup et al, 1992). We have also detected the EGFR in DMS92 by RT-PCR (data not shown). In vitro invasion was determined under serum-free conditions by a semiquantified 9-h incubation assay using Boyden chambers. Number of cells on the underside of the filters and the medium in the lower chamber was evaluated by light microscopy (LM). In vitro invasion was scored from negative (0) to positive (+++). The breast cancer cell line MDA-MB-231 was run in each experiment as a positive control (scored as +++) and in vitro invasion of SCLC cell lines was scored in relationship to this cell line. All invasion experiments were performed at least three times with similar results.

**Table 2** EGFR expression, migration and in vitro invasion in relation to Matrigel concentration

| | | | Invasion (%) | | Invasion (%) | | Invasion (%) | |
|---|---|---|---|---|---|---|---|---|
| Cell line | EGFR ($B_{max}$) | s.d. | (50 µg gel) | s.d. | (0 µg gel) | s.d. | (100 µg gel) | s.d. |
| DMS53 | 8.2 | 0.6 | 11.6 | 2.8 | 21.6 | 7.7 | 1.9 | 0.9 |
| DMS92 | – | | 16.2 | 7.8 | 48.6 | 15.4 | 6.7 | 2.5 |
| DMS114 | 5.2 | 0.5 | 5.0 | 1.4 | 22.9 | 3.4 | 2.0 | 0.9 |
| DMS273 | 3.2 | 0.9 | 13.2 | 4.2 | 32.5 | 6.0 | 7.6 | 2.0 |
| GLC2 | 28.3 | 1.9 | 8.3 | 2.1 | 50.6 | 11.5 | 3.6 | 1.8 |
| GLC3 | Neg | | 0.7 | 1.4 | 28.3 | 11.2 | 1.0 | 0.0 |
| GLC14 | Neg | | 1.8 | 0.4 | 26.6 | 1.0 | 0.2 | 0.3 |
| GLC19 | Neg | | 2.3 | 0.9 | 27.3 | 2.3 | 1.9 | 1.2 |
| NCIH69 | Neg | | 0.0 | 0.0 | 22.9 | 0.3 | 0.0 | 0.0 |
| MAR24H | 11.6 | 2.3 | 14.0 | 0.9 | 27.5 | 0.7 | 5.8 | 0.7 |
| CPH54A | 6.1 | 0.6 | 6.5 | 2.3 | 25.2 | 9.3 | 3.9 | 1.6 |
| CPH54B | 5.3 | 0.5 | 5.2 | 1.9 | 19.3 | 8.8 | 2.7 | 2.3 |
| Panel | A | | B | | C | | D | |

Maximal EGFR binding ($B_{max}$) expressed as fmol mg$^{-1}$ protein was determined at least three times, the mean values with ± s.d. are given (Panel A). In vitro invasion of eight EGFR-positive and four EGFR-negative SCLC cell lines was determined after a 24-h incubation under serum-containing conditions on Transwell filters and quantified by the MTT assay as described in Materials and methods. The Transwell filters were coated with 50 µg Matrigel. Cell number on both sides of the filters were established by the MTT assay (Panel B). For each experiment a standard curve was run and absorbance at 562 nm was used to determine cell number. In other experiments, the Transwell filters were coated with 0 (Panel C) or 100 µg (Panel D) Matrigel. Values represent the means ± s.d. of experiments performed in triplicate. Experiments were performed at least twice.

© Cancer Research Campaign 1998

**Figure 2** Migration and invasion profile of DMS92 with 0, 50 and 100 µg Matrigel. For this cell line, the in vitro invasion was performed six times by two investigators. All 18 points are given to illustrate inter- and intra-experimental variation. The box plot indicates the means, average and 5/95% confidence intervals for all data points invasion in the presence of the EGFR neutralizing monoclonal antibody mAb528. In these experiments, performed on Transwell filters, 1 µg $ml^{-1}$ mAb528 was added to the Matrigel, the apical and basal medium. Cells were cultured for 24 h in serum-free medium before the experiment and added to the serum-free medium containing 1 µg $ml^{-1}$ mAb528 at the beginning of the experiment. Cells were incubated on the Transwell filters for 24 h. An irrelevant mouse monoclonal antibody *tre*-P and mouse IgG were used as controls.

### Statistics

All experiments were performed in triplicate and values are given as means ± s.d. For evaluation of differences, student *t*-test was used. All experiments were performed at least twice.

### RESULTS

### Semiquantified in vitro invasion

We first examined all cell lines for in vitro invasion through a Matrigel membrane in a 9-h Boyden chamber invasion assay. The ability to traverse the Matrigel membrane was assessed by evaluating the number of cells on the filter together with the number of cells in the lower chamber, and relating this to the number of cells in the upper chamber. This was performed to avoid underscoring in vitro invasion of cells growing as floating aggregates, as these

**Figure 3** SCLC cell lines were cultured until confluence, at which point the medium was changed to RPMI-1640 including 1% FCS. After 4 days, the conditioned medium was collected and concentrated by freeze-drying. The redissolved material was run on a 7% SDS-PAGE gel, cast with either 1 mg $ml^{-1}$ β-casein (**A**) or gelatin (**B**), and incubated for 24 h at 37°C, stained and destained until desired contrast, between clear bands and the blue background, was obtained. Molecular weight markers (Bio-Rad) are indicated on the left. Experiments were performed twice with similar results

© *Cancer Research Campaign 1998*

**Table 3** Detection of proteolytic enzymes in SCLC cell lines

| | | Immunoprecipitation | | Western blot analysis | | |
|---|---|---|---|---|---|---|
| **Cell line** | **EGFR** | **MMP-9** | **TIMP-2** | **MMP-1** | **MMP-2** | **Cathepsin B** |
| DMS53 | + | + | + | − | − | + |
| DMS79 | + | + | + | − | − | + |
| DMS92 | + | + | + | − | − | + |
| DMS114 | + | + | + | − | − | + |
| DMS153 | + | + | + | − | − | (+) |
| DMS273 | + | + | + | − | − | + |
| DMS406 | − | + | + | − | − | + |
| DMS456 | − | + | + | − | − | − |
| GLC2 | + | + | + | − | − | (+) |
| GLC3 | − | + | + | − | − | + |
| GLC14 | − | NT | + | − | NT | NT |
| GLC16 | − | + | + | − | − | (+) |
| GLC19 | − | + | + | − | − | + |
| GLC26 | − | + | + | − | − | − |
| GLC28 | − | + | + | − | − | − |
| MAR24H | + | + | + | − | − | − |
| MAR86M1 | − | + | + | − | − | − |
| NCIH69 | − | + | + | − | − | + |
| NCI417N | + | + | + | − | − | − |
| CPH54A | + | + | + | − | − | + |
| CPH54B | + | + | + | − | − | + |

SCLC cell lines were cultured until confluence before immunoprecipitation was performed using 1 µg monoclonal antibody directed against MMP-9 or TIMP-2, and run on a 7.5% SDS page gel (MMP-9) or a 12.5–20% gradient gel (TIMP-2). In Western blot analysis (MMP-1, MMP-2 and cathepsin B), 100 µg protein was run on a 7.5% SDS-PAGE gel. Antigens were detected as described in Materials and methods. NT, Not tested. Experiments were performed twice with similar results.

**Table 4** RT-PCR detection of MMP and TIMP expression of proteolytic enzymes in SCLC cell lines

| | MMP | | | TIMP | | |
|---|---|---|---|---|---|---|
| **Cell line** | **2** | **3** | **9** | **1** | **2** | **3** |
| DMS53 | − | − | + | + | + | (+) |
| DMS79 | − | + | + | (+) | + | + |
| DMS92 | − | − | + | − | + | − |
| DMS114 | − | − | + | + | + | + |
| DMS153 | − | − | + | + | + | + |
| DMS273 | − | − | + | + | + | + |
| DMS406 | − | − | + | − | + | (+) |
| DMS456 | − | − | + | + | + | − |
| GLC2 | − | + | + | + | + | + |
| GLC3 | − | + | + | + | + | + |
| GLC14 | − | + | + | + | + | + |
| GLC16 | − | (+) | + | (+) | + | + |
| GLC19 | − | + | + | + | + | − |
| GLC26 | − | − | (+) | + | + | + |
| GLC28 | − | + | + | + | + | − |
| MAR24H | − | − | (+) | + | + | + |
| MAR86M1 | − | + | + | + | + | + |
| NC1H69 | − | + | (+) | + | + | − |
| NCI417N | − | + | + | + | + | − |
| CPH54A | − | + | + | + | + | + |
| CPH54B | − | + | + | + | + | + |

cDNA was synthesized from mRNA isolated from exponentially growing SCLC cell lines. PCR was performed as described in Materials and methods using specific oligonucleotides and run on an agarose gel. Bands were visualized by ultraviolet light. +, specific band with correct size; (+), specific but faint band; −, no band.

**Figure 4** SCLC cell lines were cultured until confluence with change of medium 24 h prior to the experiment. Cells were lysed and immunoprecipitated with 1 µg monoclonal antibody directed against MMP-9 (**A**) (GLC14 not tested) and run on a 7.5% SDS-PAGE gel or TIMP-2 (**B**) and run on a 12.5–20% SDS-PAGE gradient gel. Antigens were detected by incubating the blots with the corresponding antibodies, followed by incubation with biotinylated rabbit-anti-mouse IgG and streptavidin conjugated horseradish peroxidase. Bands were visualized by ECL, according to manufacture's instruction. In (**C**), GLC2 cells were incubated in the presence (+) or absence (−) of 1 µg of monoclonal antibody directed against TIMP-2. Bound antigens were detected as described above. Molecular weight markers (rainbow markers, Amersham) are indicated on the left. Arrowheads indicate the 92-kDa MMP-9 and the 28-kDa TIMP-2. Experiments were performed twice with similar results

 © Cancer Research Campaign 1998

**Table 5** In vitro invasion in the presence of EGFR-neutralizing mAb528

| | Addition | | | |
|---|---|---|---|---|
| **Cell line** | **None** | ***tre*-P** | **IgG** | **mAb528** |
| MAR24H | 100.0 ± 5.1 | 90.1 ± 13.4 | 114.7 ± 10.1 | 44.9 ± 3.2 |
| *P*-value | | 0.351 | 0.160 | 0.003 |
| DMS53 | 100.0 ± 11.0 | 88.2 ± 11.5 | 90.7 ± 11.8 | 41.3 ± 17.8 |
| *P*-value | | 0.245 | 0.793 | 0.002 |
| CPH54A | 100.0 ± 9.6 | 105.5 ± 2.7 | 100.9 ± 4.0 | 66.0 ± 3.3 |
| *P*-value | | 0.373 | 0.916 | 0.007 |

SCLC cell lines were seeded on Transwell filters coated with 50 μg Matrigel under serum-free conditions for 24 h. Invasion was determined by the MTT assay in the presence of the control antibody *tre*-P, mouse IgG or the EGFR neutralizing mAb528 (all 3 μg $ml^{-1}$). Invasion was normalized to the invasion in the absence of antibody. In the EGFR-negative cell lines GLC3, GLC14 and NCIH69, very low levels of invasion was found (≤ 2%). Addition of *tre*-P, mouse IgG and mAb528 did not influence these low values. Experiments were performed twice with similar results.

cells were unlikely to be attached to the underside of the filters. The breast cancer cell line MDA-MB-231 has a high number of EGFR and a high degree of in vitro invasion (Fitzpatrick et al, 1984; Long and Rose, 1996). In all experiments, this cell line was used as a positive control and the invasion was scored as +++. The invasion of SCLC cell lines was then related to the invasion of MDA-MB-231. This in vitro invasion was scored from negative 0 to +++. No invasion was scored as 0, few cells as +, numerous cells as ++ and a similar number of invading SCLC cells to that of MDA-MB-231 as +++. The results are summarized in Table 1. The scoring was performed by two of the authors (LD and NB, the latter without the knowledge of EGFR status). The filters were stained and photographed as seen in Figure 1, in which the in vitro invasion of 2 SCLC cell lines and MDA-MB-231 cell line is illustrated. Based on these data, we could divide our panel of SCLC cell lines into two groups: cells with an ability to cross the Matrigel membrane and cells without this ability. From Table 1, it can be seen that all the EGFR-positive cell lines had in vitro invasive capability. It is also shown that all the EGFR-negative cell lines did not traverse the Matrigel membrane. Furthermore, it appears that the in vitro invasion did not correlate with the growth characteristics of the SCLC cell lines, i.e. cells growing as floating aggregates vs monolayer cultures.

### Quantified in vitro invasion

To quantify in vitro invasion, we selected eight EGFR-positive and four EGFR-negative SCLC cell lines (Table 2, A). These cell lines were tested in an invasion assay with a longer incubation time (24 h). Invasion was performed on Transwell filters under serum-containing conditions. As illustrated in Table 2 B, the degree of invasion in EGFR-positive cell lines ranged from 5.0% ± 1.4% (DMS114) to 16.2% ± 7.8% (DMS92), whereas in the EGFR-negative SCLC cell lines tested less than 2.3% of the cells traversed the Matrigel membrane. These results therefore agree with the results from the in vitro invasion based on light microscopy.

### In vitro invasion with different Matrigel concentrations

To assess whether the inability of the EGFR-negative cell lines to traverse the Matrigel membrane was due to an impaired migration/motility capability, we performed the in vitro 'invasion' in the absence of Matrigel. Furthermore, to evaluate the fidelity of the Matrigel membrane, we also performed the assay using 100 μg Matrigel/filter. For these studies, we selected the same EGFR-positive and -negative SCLC cell lines as above. We found that, in the examined SCLC cell lines, migration/motility was between 19.3% and 50.6% in the eight examined EGFR-positive cell lines and between 22.9% and 28.3% in the four EGFR-negative cell lines (Table 2 C). This indicates that non-invasive SCLC cell lines did not have a faulty motility ability, suggesting that the EGFR-negative cell lines had a lower or defective ability to degrade the Matrigel membrane. Furthermore, we found that the in vitro invasion in EGFR-positive SCLC cell lines was significantly lower in experiments using 100-μg compared with 50 μg Matrigel/filter (Figure 2 and Table 2 D). These results, as well as the lack of in vitro invasion in EGFR-negative cell lines, indicated that the Matrigel membrane forms a physical barrier, which has to be degraded before cells appear in the lower compartment.

### Proteolytic activity of SCLC cell lines

Our finding that the Matrigel membrane formed a physical barrier which had to be degraded indicated that SCLC cell lines secreted proteolytic enzymes enabling the degradation of the Matrigel membrane. To determine the proteolytic activity of the SCLC cell lines, zymography of conditioned medium was performed. In Figure 3, the results of a zymogram are shown. It can be seen that SCLC cell lines express a variety of enzymes able to degrade β-casein (Figure 3A). In other experiments, all SCLC cell lines were found to express gelatine-degrading proteases (Figure 3B). To explore these proteolytic enzymes further, we examined selected proteases by immunoprecipitation, Western blot analysis and RT-PCR. In immunoprecipitation studies, we examined MMP-9 and TIMP-2 (Table 3). A representative blot for MMP-9 is seen in Figure 4A, showing that all the 20 examined SCLC cell lines expressed the 92-kDa protein MMP-9. Figure 4B shows a TIMP-2 immunoprecipitation blot. The specificity of the TIMP-2 immunoprecipitation in GLC2 is seen in Figure 4C. Western blot analysis was used to analyse the presence of MMP-1, MMP-2 and cathepsin B; these results are shown in Table 3. In Table 4, the results from RT-PCR using specific oligonucleotides to detect MMP-2, MMP-3, MMP-9, TIMP-1, TIMP-2 and TIMP-3 are summarized. Our results from the zymograms, immunoprecipitation and Western blot analysis and RT-PCR studies indicate that SCLC cell lines expressed a variety of proteolytic enzymes and inhibitors. However, a pattern of expression of these molecules that could distinguish in vitro invasive SCLC cell lines from non-invasive was not apparent.

### In vitro invasion in the presence of EGFR neutralizing mAb

Our results raised the question of whether the EGFR was involved in the in vitro invasive phenotype of the examined SCLC cell lines. To address this question, we performed quantified in vitro invasion in the presence of the EGFR-neutralizing mouse monoclonal antibody mAb528 using Transwell filters. As a control, these experiments were also performed in the presence of the irrelevant mouse monoclonal antibody *tre*-P or mouse IgG. For these experiments, we selected six cell lines: the EGFR-negative SCLC cell lines GLC3, GLC14 and NCIH69 and the three EGFR-positive SCLC cell lines MAR24H, DMS53 and CPH54A. For comparison, the results were normalized to the invasion in the absence of antibody. We show that in vitro invasion in the three EGFR-positive cell lines was reduced significantly after mAb528 addition to 44.9% (MAR24H), 41.3% (DMS53) and 66.0% (CPH54A) ($P$-values = 0.003, 0.002 and 0.007 respectively). Experiments performed in the presence of *tre*-P or mouse IgG did not influence invasion, indicating that the EGFR was directly involved in the in vitro invasive phenotype of SCLC cell lines (Table 5). In the three EGFR-negative SCLC cell lines, no effect was observed (data not shown).

### DISCUSSION

In this study, we have examined a panel of SCLC cell lines for their ability to traverse a reconstituted Matrigel membrane and related this in vitro invasion to the EGFR status. Of the 21 SCLC cell lines in our panel, 11 cell lines were characterized as EGFR positive based on Northern blot analysis, chemical cross-linking and Scatchard analysis of the binding data and ten cell lines as EGFR-negative (Damstrup et al, 1992). All cell lines were analysed in a Boyden chamber assay and the in vitro invasion was semiquantified by comparing the invasion of SCLC cell lines with MDA-MB-231 cells. We show that all 11 EGFR-positive SCLC cell lines were invasive, whereas none of the EGFR-negative cell lines had the ability to traverse the reconstituted Matrigel membrane (Table 1). This difference in in vitro invasion was confirmed in the quantified invasion assay. However, the level of in vitro invasion was independent of growth characteristics and EGFR expression level (Tables 1 and 2). In other cell types, a role for the EGFR has also been demonstrated, Hölting et al, (1995) have shown that in a follicular thyroid cancer cell line EGF or transforming growth factor $\alpha$ (TGF$\alpha$) stimulated invasion over unstimulated cells ($P < 0.02$). Similar results were found by Hamada et al (1995) in rat mammary carcinoma cells, in which EGF, in a dose-dependent manner, stimulated in vitro invasion. Thus, our results support the notion that EGFR has a role in the invasive phenotype.

Examination of the motility/migration of the cell lines showed that all examined SCLC cell lines in this assay had a similar high degree of motility, irrespective of EGFR expression. Hence, in EGFR-negative SCLC cell lines the Matrigel membrane formed a physical barrier that could not be traversed, whereas EGFR-positive SCLC cell lines had the ability to degrade the Matrigel membrane.

The examined SCLC cell lines expressed a variety of proteolytic enzymes, which could degrade the Matrigel membrane. However, we could not differentiate between in vitro invasive and non-invasive SCLC cell lines based on the expression pattern of the examined proteases. Furthermore, in a preliminary study of four SCLC cell lines in our panel, no difference in protein expression of uPA, urokinase-type plasminogen activator receptor (uPAR) or plasminogen activator inhibitor (PAI-1) was found among the two examined EGFR-positive and the two EGFR-negative cell lines (L Damstrup et al, unpublished observation). Others have found that growth factors belonging to the EGF-like family of ligands could stimulate different systems involved in the degradation of basement membrane. Harvey et al (1995) have shown that EGF was able to stimulate the production of MMP-9 in blastocyst outgrowth. Yoshida et al (1990) have shown that EGF and TGF-$\alpha$ increased expression of MMP-3 and MMP-7 in a human gastric carcinoma cell line. Lund et al (1995) have shown that EGF stimulation of the lung cancer cell line A549 resulted in increased expression of uPA. These results suggest that the EGFR, at least to some extent, can be involved in the invasive phenotype by regulating the production of proteolytic enzymes capable of degrading crucial basement membrane components.

The observation that TIMP-2 was expressed by in vitro invasive SCLC cell lines raised the question why invasive SCLC cell lines expressed an inhibitor of a proteolytic enzyme. An explanation might be that TIMP-2 increases the stability of the 72 kDa type IV collagenase by preventing autocatalytic activation and degradation (Howard et al, 1991; Kleiner et al, 1993).

All SCLC cell lines in our panel express one or more of the ligands that binds to the EGFR (L Damstrup et al, unpublished observation) and, to address the role of endogenously produced ligands binding to the EGFR, we examined the in vitro invasion in the presence of the EGFR-neutralizing mAb528. We showed that mAb528 significantly reduced the in vitro invasion in three EGFR-positive cell lines, whereas no alteration in in vitro invasion was seen after the addition of control mAb or mouse IgG. Our results suggest that the positive SCLC cell lines produced biologically active ligands that bound to the EGFR in an autocrine fashion. Furthermore, our results suggest that this ligand– receptor binding may influence the production of proteases involved in the in vitro invasion.

More direct evidence for an involvement of EGFR in the invasive phenotype has been reported by Lichtner et al (1995). They examined two clones of the rat mammary tumour 13762NF. One clone expressed high levels of EGFR and had a high incidence of lung metastasis. The other clone expressed low levels of EGFR and formed few metastases. After stable transfection with EGFR, the low-EGFR-expressing clone had a higher incidence of metastasis. Xie et al (1995) transfected DU-145, human prostate carcinoma cells, with full-length EGFR and showed that the transfected cells had a 1.8-fold increased invasion in human amniotic basement membrane matrix compared with the parental cell line. We have recently, in two *lacZ*-transfected SCLC cell lines, found formation of metastasis in *nu/nu*-META/Bom mice. In GLC2 metastasis was seen frequently, whereas in DMS456 metastasis was seen only in a few cases (Rømer et al, 1995). Our observation that only EGFR-positive SCLC cell lines were invasive in the in vitro system are thus supported by these in vivo data.

 © Cancer Research Campaign 1998

In conclusion, we have demonstrated that in our panel of 21 SCLC cell lines in vitro invasion was strongly correlated with the presence of the EGFR. We also demonstrate that all SCLC cell lines expressed several enzymes able to degrade components of the basement membrane. However, an expression pattern among EGFR-positive or EGFR-negative SCLC cell lines could not be established, indicating that several regulatory mechanisms are involved in conferring in vitro invasion in SCLC cell lines.

### ACKNOWLEDGEMENTS

This work was supported by the Danish Cancer Society grant no. 93-031, the Danish Research Academy, the Danish Research Council grant no. 9305111, the Danish Society for Cancer Research, the Haench, the Henriksen, the Madsen and the Vissing Foundations.

### REFERENCES

Albini A, Kleinman HK, Martin GR, Aaronson SA, Kozlowski JM and McEwan RN (1987) A rapid in vitro assay for quantitating the invasive potential of tumor cells. *Cancer Res* **47**: 3239–3245

Bepler G, Jaques G, Neumann K, Aumüller G, Gropp C and Havemann K (1987) Establishment, growth properties, and morphological characteristics of permanent human small cell lung cancer cell lines. *J Cancer Res Clin Oncol* **113**: 31–40

Berendsen HH, De Leij L, De Vries EGE, Mesander G, Mulder NH, De Jong B, Buys CHCM, Postmus PE, Poppema S, Sluiter HJ and The HT (1988) Characterization of three small cell lung cancer cell lines established from one patient during longitudinal follow-up. *Cancer Res* **48**: 6891–6899

Carney DN, Gazdar AF, Bepler G, Guccion JG, Marangos PJ, Moody TW, Zweig MH and Minna JD (1985) Establishment and identification of small cell lung cancer cell lines having classic and variant features. *Cancer Res* **45**: 2913–2923

Damstrup L, Rygaard K, Spang-Thomsen M and Skovgaard Poulsen H (1992) Expression of the epidermal growth factor receptor in human small cell lung cancer cell lines. *Cancer Res* **52**: 3089–3093

De Leij L, Postmus PE, Buys CHCM, Elema JD, Ramaekers F, Poppema S, Brouwer M, Van der Veen AY, Mesander G and The TH (1985) Characterization of three new variant type cell lines derived from small cell carcinoma of the lung. *Cancer Res* **45**: 6024–6033

De Wit PEJ, Moretti S, Koenders PG, Weterman MAJ, Van Muijen GNP, Gianotti B and Ruiter DJ (1992) Increasing epidermal growth factor receptor expression in human melanocytic tumor progression. *J Invest Dermatol* **99**: 168–173

Dignass AU, Tsunekawa S and Podolsky DK (1994) Fibroblast growth factors modulate intestinal epithelial cell growth and migration. *Gastroenterology* **196**: 1254–1262

Engelholm SA, Spang-Thomsen M, Vindeløv LL, Brünner N, Nielsen MH, Hirsch F, Nielsen A and Hansen HH (1986) Comparison of characteristics of human small cell carcinoma of the lung in patients, in vitro and transplanted into nude mice. *Acta Pathol Microbiol Immunol Scand* **94**: 325–336

Fidler IJ and Nicolson GL (1987) The process of cancer invasion and metastasis. *Cancer Bull* **39**: 126–131

Fitzpatrick SL, Lachance MP and Schultz GS (1984) Characterization of epidermal growth factor receptor and action on human breast cancer cells in culture. *Cancer Res* **44**: 3442–3447

Hamada J, Nagayasu H, Takayama M, Kawano T, Hosokawa M and Takeichi N (1995) Enhanced effect of epidermal growth factor on pulmonary metastasis and in vitro invasion of rat mammary carcinoma cells. *Cancer Lett* **89**: 161–167

Harvey MB, Leco KJ, Arcellana-Panlilio MY, Zhang X, Edwards DR and Schultz GA (1995) Proteinase expression in early mouse embryos is regulated by leukaemia inhibitory factor and epidermal growth factor. *Development* **121**: 1005–1014

Heussen C and Dowdle EB (1980) Electrophoretic analysis of plasminogen activators in polyacrylamide gels containing sodium dodecyl sulfate and copolymerized substrates. *Anal Biochem* **102**: 196–202

Hollas W, Blasi F and Boyd D (1991) Role of the urokinase receptor in facilitating extracellular matrix invasion by cultured colon cancer. *Cancer Res* **51**: 3690–3695

Hölting T, Siperstein AE, Clark OH and Duh QY (1994) Epidermal growth factor enhances proliferation, migration, and invasion of follicular and papillary thyroid cancer in vitro and in vivo. *J Clin Endocrinol Metab* **79**: 401–408

Hölting T, Siperstein AE, Clark OH and Duh QY (1995) Epidermal growth factor (EGF)- and transforming growth factor alpha-stimulated invasion and growth of follicular thyroid cancer cells can be blocked by antagonism to the EGF receptor and tyrosine kinase in vitro. *Eur J Endocrinol* **132**: 229–235

Hoosein NM, Boyd D, Hollas W, Mazar A, Henkin J and Chung LWK (1991) Involvement of urokinase and its receptor in the invasiveness of human prostatic carcinoma cell lines. *Cancer Commun* **3**: 255–264

Howard EW, Bullen EC and Banda MJ (1991) Regulation of autoactivation of human 72-kDa progelatinase by tissue inhibitor of metalloproteinases-2. *J Biol Chem* **266**: 13064–13069

Jasonni VM, Amadori A, Santini D, Ceccarelli C, Naldi S and Flamigni C (1995) Epidermal growth factor receptor (EGF-R) and transforming growth factor alpha (TGFα) expression in different endometrial cancers. *Anticancer Res* **15**: 1327–1332

Kleiner Jr DE, Tuuttila A, Tryggvason K and Stetler-Stevenson WG (1993) Stability analysis of latent and active 72-kDA type IV collagenase: the role of tissue inhibitor of metalloproteinases-2 (TIMP-2). *Biochemistry* **32**: 1583–1592

Kohga S, Shashikumar RH, Weaver RM and Markus G (1985) Localization of plasminogen activators in human colon cancer by immunoperoxidase staining. *Cancer Res* **45**: 1787–1798

Liabakk N, Talbot I, Smith RA, Wilkinson K and Balkwill F (1996) Matrix metalloprotease 2 (MMP-2) and matrix metalloprotease 9 (MMP-9) type IV collagenases in colorectal cancer. *Cancer Res* **56**: 190–196

Lianos S, Iglesias T, Riese HH, Garrido T, Caelles C and Muñoz A (1996) v-*erb*A oncogene induces invasiveness and anchorage-independent growth in cultured glial cells by mechanisms involving platelet-derived growth factor. *Cell Growth Different* **7**: 373–382

Lichtner RB, Kaufmann AM, Kittmann A, Rohde Schulz B, Walter J, Williams L, Ullrich A, Schirrmacher V and Khazaie K (1995) Ligand mediated activation of ectopic EGF receptor promotes matrix protein adhesion and lung colonization of rat mammary adenocarcinoma cells. *Oncogene* **10**: 1823–1832

Liotta LA, Goldfarb R, Brundage R, Siegal G, Terranova V and Garbisa S (1991) Effects of plasminogen activator (urokinase), plasmin, and thrombin, glycoprotein and collagenous components of basement membrane. *Cancer Res* **41**: 4629–4636

Long BJ and Rose DP (1996) Invasive capacity and regulation of urokinase-type plasminogen activator in estrogen receptor (ER)-negative MDA-MB-231 human breast cancer cells, and a transfectant (S30) stably expressing ER. *Cancer Lett* **99**: 209–215

Lund LR, Ellis V, Rønne E, Pyke C and Danø K (1995) Transcriptional and post-transcriptional regulation of the receptor for urokinase-type plasminogen activator by cytokines and tumour promoters in human lung carcinoma cell line A549. *Biochem J* **310**: 345–352

Lund-Johansen M, Forsberg K, Bjerkvig R and Laerum OD (1992) Effects of growth factors on a human glioma cell line during invasion into rat brain aggregates in culture. *Acta Neuropathol* **84**: 190–197

Marcotte PA, Kozan IM, Dorwin SA and Ryan JM (1992) The matrix metalloproteinase pump-1 catalyzes formation of low molecular weight (pro)urokinase in cultures of normal human kidney cells. *J Biol Chem* **267**: 13803–13806

Markus G, Camiolo SM, Kohga S, Madeja JM and Mittelman A (1983) Plasminogen activator secretion of human tumors in short-term organ culture, including a comparison of primary and metastatic colon tumors. *Cancer Res* **43**: 5517–5525

McDonnell S and Matrisian LM (1990) Stromelysin in tumor progression and metastasis. *Cancer Metastasis Rev* **9**: 305–319

McDonnell S, Navre M, Coffey RJ, Jr and Matrisian LM (1991) Expression and localization of the matrix metalloproteinase Pump-1 (MMP-7) in human gastric and colon carcinomas. *Mol Carcinogen* **4**: 527–533

Neal D, Bennett MK, Hall RR, Marsh C, Abel PD, Sainsbury JRC and Harris AL (1985) Epidermal-growth factor receptors in human bladder cancer: comparison of invasive and superficial tumours. *Lancet* **i**: 366–368

Nishida T, Nakamura M, Murakami J, Mishima H and Otori T (1992) Epidermal growth factor stimulates corneal epithelial cell attachment to fibronectin through a fibronectin receptor system. *Invest Ophthalmol Vis Sci* **33**: 2464–2469

Okada Y, Gonoji Y, Naka K, Tomita K, Nakanishi I, Iwata K, Yamashita K and Hayakawa T (1992) Matrix metalloproteinase-9 (92-kDa gelatinase/type-IV collagenase) from HT-1080 human fibrosarcoma cells: purification and activation of the precursor and enzymic properties. *J Biol Chem* **267**: 21712–21719

© Cancer Research Campaign 1998

Ossowski L, Clunie G, Masucci M-T and Blasi F (1991) In vivo paracrine interaction between urokinase and its receptor: effect on tumor cell invasion. *J Cell Biol* **115:** 1107–1112

Pettengill OS, Sorenson GD, Wurster-Hill D, Curphey TJ, Noll WW, Cate CC and Maurer LH (1980) Isolation and growth characteristics of continuous cell lines from small-cell carcinoma of the lung. *Cancer* **45:** 906–918

Reith A and Rucklidge GJ (1992) Invasion of brain tissue by primary glioma: evidence for the involvement of urokinase-type plasminogen activator as an activator of type-IV collagenase. *Biochem Biophys Res Commun* **186:** 348–354

Rømer MU, Christiansen J, Brünner N and Spang-Thomsen M (1995) Dissemination in athymic nude mice of *lacZ* transfected small cell lung cancer cells identified by X-gal staining. *Acta Pathol Microbiol Immunol Scand* **103:** 582–587

Salamonsen LA, Nagase H and Woolley DE (1991) Production of matrix metalloproteinase 3 (stromelysin) by cultured ovine endometrial cells. *J Cell Sci* **100:** 381–385

Sappino A-P, Busso N, Belin D and Vassalli J-D (1987) Increase of urokinase-type plasminogen activator gene expression in human lung and breast carcinoma. *Cancer Res* **47:** 4043–4046

Shibamoto S, Hayakawa M, Hori T, Oku N, Miyazawa K, Kitamura N and Ito F (1992) Hepatocyte growth factor and transforming growth factor β stimulate both cell growth and migration of human gastric adenocarcinoma cells. *Cell Struct Funct* **17:** 185–190

Skriver L, Larsson LI, Kielberg V, Nielsen LS, Andresen PI and Danø K (1984) Immunocytochemical localization of urokinase type plasminogen activator in Lewis lung carcinoma. *J Cell Biol*, **99:** 751–757

Sreenath T, Matrisian LM, Stetler-Stevenson W, Gattoni-Celli S and Pozzatti RO (1992) Expression of matrix metalloproteinase genes in transformed rat cell lines of high and low metastatic potential. *Cancer Res* **52:** 4942–4947

Testa JE (1992) Loss of the metastatic phenotype by a human epidermoid carcinoma cell line, HEp-3, is accompanied by increased expression of tissue inhibitor of metalloproteinase 2. *Cancer Res* **52:** 5597–5603

Tryggvarson K, Hoyhtya M and Salo T (1987) Proteolytic degradation of extracellular matrix in tumor invasion. *Biochim Biophys Acta* **907:** 191–210

Veale D, Ashcroft T, Marsh C, Gibson GJ and Harris AL (1987) Epidermal growth factor in non-small cell lung cancer. *Br J Cancer* **55:** 513–516

Vistica DT, Skehan P, Scudiero D, Monks A, Pittman A and Boyd MR (1991) Tetrazolium-based assays for cellular viability: a critical examination of selected parameters affecting formazan production. *Cancer Res* **51:** 2515–2520

Wun T, Schleuning W and Reich E (1982) Isolation and characterization of urokinase from human placenta. *J Biol Chem* **257:** 3276–3283

Xie H, Turner T, Wang MH, Singh RK, Siegal GP and Wells A (1995) In vitro invasiveness of DU-145 human prostate carcinoma cells is modulated by EGF receptor-mediated signals. *Clin Exp Metastasis* **13:** 407–419

Yano H, Shiozaki H, Kobayashi K, Yano T, Tahara H, Tamura S and Mori T (1991) Immunohistologic detection of epidermal growth factor receptor in human esophageal squamous cell carcinoma. *Cancer* **67:** 91–98

Yasui W, Hata J, Yokozaki H, Nakatani H, Ochiai A, Ito H and Tahara E (1988) Interaction between epidermal growth factor and its receptor in progression of human gastric carcinoma. *Int J Cancer* **41:** 211–217

Yoshida K, Tsujino T, Yasui W, Kameda T, Sano T, Nakayama H, Toge T and Tahara E (1990) Induction of growth factor-receptor and metalloproteinase genes by epidermal growth factor and/or transforming growth factor-α in human gastric carcinoma cell line MKN-28. *Jpn J Cancer Res* **81:** 793–798

© Cancer Research Campaign 1998